# Immediate and delayed antibiotic prescribing strategies used by Australian early-career GPs: a cross-sectional analysis

**DOI:** 10.3399/BJGP.2021.0026

**Published:** 2021-11-02

**Authors:** Andrew Davey, Amanda Tapley, Katie J Mulquiney, Mieke van Driel, Alison Fielding, Elizabeth Holliday, Joshua S Davis, Paul Glasziou, Anthea Dallas, Jean Ball, Neil Spike, Kristen FitzGerald, Parker Magin

**Affiliations:** NSW & ACT Research and Evaluation Unit, GP Synergy, Regional Training Organisation, Australia.; NSW & ACT Research and Evaluation Unit, GP Synergy, Regional Training Organisation, Australia.; NSW & ACT Research and Evaluation Unit, GP Synergy, Regional Training Organisation, Australia.; Faculty of Medicine, University of Queensland, Brisbane, Australia.; NSW & ACT Research and Evaluation Unit, GP Synergy, Regional Training Organisation, Australia.; School of Medicine and Public Health, University of Newcastle, Australia.; Menzies School of Health Research, Darwin, Australia.; Institute for Evidence-Based Healthcare, Bond University, Robina, Australia.; Tasmanian School of Medicine, University of Tasmania, Hobart, Australia.; Hunter Medical Research Institute, New Lambton Heights, Australia.; Eastern Victoria General Practice Training, Regional Training Organisation, Hawthorn, Australia.; General Practice Training Tasmania, Regional Training Organisation, Hobart, Australia.; School of Medicine and Public Health, University of Newcastle, Australia.

**Keywords:** antibiotics, family practice, general practice, primary health care, respiratory tract infections

## Abstract

**Background:**

Antibiotics are overused for non-pneumonia acute respiratory tract infections (ARTIs).

**Aim:**

To establish prevalence and explore associations of delayed and immediate antibiotic prescribing strategies of Australian early-career GPs (specialist GP vocational trainees, also known as GP registrars) for non-pneumonia ARTIs.

**Design and setting:**

Cross-sectional analysis of data collected between September 2016 and December 2017 from the Registrar Clinical Encounters in Training cohort (ReCEnT) study, an ongoing cohort study of GP registrars’ in-practice clinical experiences in four Australian states and territories.

**Method:**

Multinomial logistic regression with outcome antibiotic prescribing (no prescribing, immediate prescribing, and delayed prescribing).

**Results:**

Of 7156 new ARTI diagnoses, no antibiotics were prescribed for 4892 (68%); antibiotics were prescribed for immediate use for 1614 diagnoses (23%) and delayed antibiotics were used for 650 diagnoses (9%). Delayed prescribing was used in 22% of otitis media, 16% of sinusitis, 13% of sore throat, 11% of acute bronchitis/bronchiolitis, and 5% of upper respiratory tract infection (URTI) diagnoses. Delayed prescribing was used for 29% of all prescriptions written. Delayed prescribing and immediate prescribing were associated with markers of clinical concern. Delayed prescribing was associated with longer duration of consultation and with fewer diagnoses/problems dealt with in the consultation.

**Conclusion:**

Australian early-career GPs use no prescribing for ARTIs substantially more than established GPs; however, except where URTIs are concerned, they still prescribe antibiotics in excess of validated benchmarks. Australian early-career GPs may use delayed prescribing more often than European established GPs, and may use it to manage diagnostic uncertainty and, possibly, conflicting influences on prescribing behaviour. The use of delayed prescribing may enable a transition to an environment of more-rational antibiotic prescribing for ARTIs.

## INTRODUCTION

Antibiotic resistance and rational antibiotic use are national priorities in Australia, but antibiotics remain overused for non-pneumonia acute respiratory tract infections (ARTIs),^1^^,^^2^ which are common presentations to GPs.^3^ In 2017, 41.5% (*n* = 10 215 109) of the Australian population was dispensed at least one systemic antibiotic in primary care, and Australia is in the highest 25% of countries (European countries and Canada) in terms of rates of community antibiotic use.^2^^,^^4^ When managing ARTIs, antibiotics are mostly not indicated.^1^

Doctors’ use of antibiotics can be classified as: no prescribing, delayed prescribing, and immediate prescribing. Delayed prescribing is when the intention is to not use antibiotics unless certain criteria are met (for example, a deterioration in condition). A Cochrane review has shown that, when compared with immediate prescribing, no prescribing and delayed prescribing significantly reduce consumption of inappropriate antibiotics for ARTIs, with no difference in patient satisfaction between delayed prescribing and immediate prescribing.^5^

Some drivers of prescribing antibiotics for ARTIs are diagnostic uncertainty and concern about the consequences of potential serious illness.^6^^,^^7^ However, this concern may be misplaced because both delayed prescribing and no prescribing appear to be safe, with no statistically significant difference in hospital admissions or death when compared with immediate prescribing in a UK cohort of almost 29 000 people.^8^ Additionally, the study showed that delayed prescribing led to a significant reduction in re-consultation for new, worsening, or non-resolving symptoms, compared with both no prescribing and immediate prescribing.^8^ Although there is ample literature — including a Cochrane review^5^ — of trials of delayed prescribing, there is little evidence for the use of delayed prescribing in practice.^9^

General practice training is an important time for early-career GPs, during which they develop approaches to diagnosing and managing ARTIs, including antibiotic prescribing practices. There is evidence from a qualitative study that GPs’ decision-making processes regarding antibiotic prescribing are consistent over time.^10^

In the study presented here, the authors aimed to:

- describe the prevalence of antibiotic prescribing strategies (immediate prescribing, delayed prescribing, and no prescribing) used by Australian early-career GPs (specialist GP vocational trainees, also known as GP registrars) for initial presentations of non-pneumonia ARTIs; and
- establish the determinants of registrars’ choice of prescribing strategy.

**Table:** How this fits in

In Australia, GPs prescribe antibiotics for acute respiratory tract infections (ARTIs) well in excess of validated benchmarks. Australian early-career GPs (specialist GP vocational trainees, also known as GP registrars [13% of the GP workforce]) use no prescribing of antibiotics for ARTIs substantially more often than established Australian and European GPs, but still prescribe in excess of benchmarks, except for upper respiratory tract infections. They appear to use delayed prescribing of antibiotics to manage diagnostic uncertainty and, perhaps also, conflicting influences on antibiotic prescribing for ARTIs. Delayed prescribing may facilitate the transition to a culture of more-rational antibiotic prescribing for ARTIs in Australia.

## METHOD

### Participants

This study comprised a cross-sectional analysis of data from the Registrar Clinical Encounters in Training (ReCEnT) cohort study.^11^ Data were collected from September 2016 until December 2017; the study methodology has been described in detail elsewhere.^11^ Briefly, ReCEnT is an ongoing cohort study of GP registrars’ in-practice clinical experiences undertaken in four of the eight Australian states and territories. ReCEnT is a component of GP registrars’ educational programme, facilitating reflection on practice and learning.^12^^,^^13^ Registrars may consent to their data being used for research purposes.

### Procedures

Characteristics of participating GP registrars and their practices are documented via a questionnaire at the beginning of each 6-monthly general practice-based training term (three compulsory terms). Registrars then record 60 consecutive office-based consultations (approximately 1 week of consultations) at approximately the midpoint of each term.

Problems/diagnoses are coded according to the International Classification of Primary Care, second edition (ICPC-2).^14^ ARTI diagnoses were defined by the following codes: R74 (upper respiratory tract infection [URTI]; R78 (acute bronchitis/bronchiolitis); R75 (acute sinusitis); H71 (acute otitis media); R72 (strep throat); R76 (tonsillitis, acute); R74008 (pharyngitis, acute); R74006 (infection, throat); R74017 (pharyngitis); and R21005 (sore throat). R72, R76, R74008, R74006, and R74017 were grouped together as ‘sore throat’ for analysis.

Medications prescribed, provided in-consultation, or recommended are routinely recorded. Antibiotic medications were identified using the Anatomic Therapeutic Chemical (ATC) classification.^15^

GP registrars reported whether delayed or immediate prescribing was employed for diagnoses involving the prescription of antibiotics. No prescribing was defined as the absence of an antibiotic prescription for an ARTI diagnosis. GP registrars recorded a delayed prescription when they asked the patient not to start the antibiotic immediately.

### Outcome factors

The primary outcome factor was whether a new ARTI diagnosis (that is, a diagnosis for a first presentation — re-consultations were excluded from analysis) involved delayed, immediate, or no prescribing.

### Independent variables

After consideration of the potential relevance of individual variables to the research question/outcome factor (including considerations of the literature, the authors’ clinical experience, and their experience with this study population and this dataset), the analysis plan, a priori, included 24 variables that were deemed relevant. These variables related to the patient, GP registrar, practice, and the consultation. Some require explanation:

- routinely bulk bills — there is no financial cost to the patient for the consultation;
- rurality — practice postcode was used to define the practice’s Australian Standard Geographical Classification-Remoteness Area classification;^16^
- socioeconomic status of the practice location — classified according to the Socioeconomic Index for Areas Index of Relative Disadvantage^17^ of the practice location;
- sought clinical information or assistance during the consultation — help was sought from the GP supervisor, a specialist, or electronic or hard-copy resources; and
- learning goals generated — clinical questions to be pursued after the consultation had finished.

### Data analysis

The proportions of all ARTI diagnoses involving delayed, immediate, and no prescribing were calculated with 95% confidence intervals (CIs); they were also calculated by type of ARTI.

Multinomial logistic regression for a nominal outcome was used within the generalised estimating equations (GEE) framework to account for repeated measures within GP registrars. GEE was used for analyses, as the authors’ interest was in effect estimates averaged across registrars, rather than registrar-specific effects (as produced with mixed [random-effects] models). To estimate the GEE, an exchangeable working correlation structure was assumed.

Multinomial logistic regression for a nominal outcome with three (non-ordered) levels produces two sets of statistics: one for each of the two test levels compared with the reference level. Delayed prescribing was used as the reference level. The model odds ratios (ORs) compared no prescribing with delayed prescribing, and immediate prescribing with delayed prescribing. The results are equivalent to conducting two binary logistic models.

Covariates with a univariate *P*-value of <0.20 were considered for inclusion in the multiple regression model. For each outcome, a model with all selected covariates was fitted, after which model reduction was assessed. Covariates that were no longer significant (at *P*<0.20) in the multivariable model were tested for removal from the model. If the covariate’s removal did not substantively change the remaining coefficients in the model by >∼10%, the covariate was removed from the final model.

In a post-hoc analysis, association of delayed prescribing, immediate prescribing, or no prescribing with seeking assistance from the registrar’s supervisor was tested using a χ^2^ statistic.

Analyses were programmed using Stata (version 14.0) and SAS (version 9.4).

## RESULTS

A total of 631 GP registrars completed 1063 rounds of data collection (*N* = 1092, response rate 97.3%) at 454 practices contributed 101 019 patient problems/diagnoses from 63 628 consultations. The characteristics of participating GP registrars and their practices are shown in Table 1.

**Table 1.** Characteristics of GP registrars and their teaching practices

**Registrar variables, *N* = 631**	***n* (%)^a^**
Sex, Female	384 (61)

Qualified as doctor overseas	77 (12)

Years worked in hospital before entering GP training, mean (SD)	3.0 (2.6)

**Registrar round/practice variables, *N* = 1070^b^**	

Worked at the practice in a previous term	224 (21)

Registrar age, years, mean (SD)	32.5 (6.3)

Registrar works part time	223 (22)

Registrar training term	
Term 1	414 (39)
Term 2	458 (43)
Term 3	198 (19)

Practice rurality/urbanicity	
Major city	728 (69)
Inner regional	237 (22)
Outer regional	94 (9)
Remote	0 (0)
Very remote	0 (0)

Practice location SES status (SEIFA index), mean (SD)	5.8 (2.9)

Practice routinely bulk bills	367 (36)

Full-time equivalent GPs working at the practice	
1–4	426 (41)
≥5	602 (59)

a *Unless otherwise stated.*

b *Numbers may not add up to 1070 due to missing data. SD = standard deviation. SEIFA = Socioeconomic Index for Areas. SES = socioeconomic status.*

**Figure 1.**
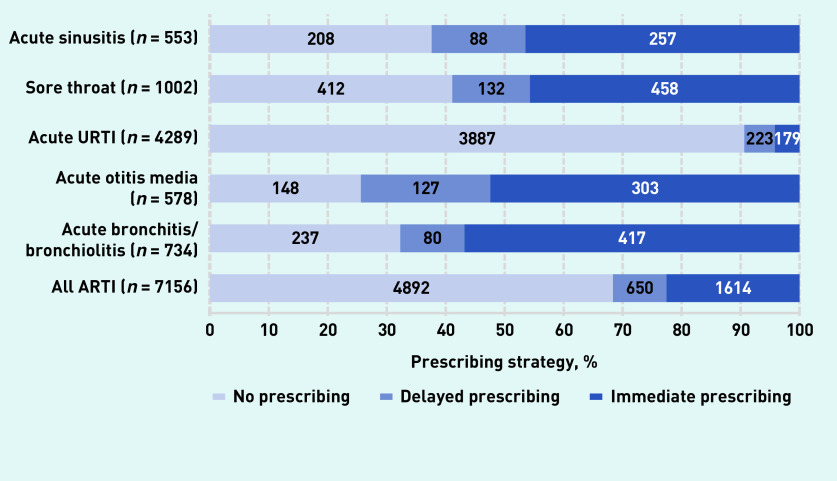
*Frequency of antibiotic prescribing strategies.*

There were 7156 new diagnoses of an ARTI; of these, no antibiotics were prescribed for 4892 (68% [95% CI = 67% to 69%]) diagnoses, antibiotics were prescribed for immediate use for 1614 (23% [95% CI = 22% to 24%]) diagnoses, and delayed antibiotics were used for 650 (9% [95% CI = 8% to 10%]) diagnoses (see Figure 1 and Supplementary Table S1 for details of each ARTI diagnosis). When prescribing antibiotics for ARTIs, GP registrars used delayed prescribing 29% of the time.

Characteristics associated with ARTI diagnoses by antibiotic prescribing strategy are presented in Table 2.

**Table 2.** Antibiotic prescribing for acute respiratory tract infections^a^

**Variable**	**Total^b^**	**No prescribing^b^**	**Immediate prescribing^b^**	**Delayed prescribing^b^**	***P*-value**
**Age, years**					
0− <5	1504 (21)	1119 (23)	258 (16)	127 (20)	<0.001
5–14	1067 (15)	722 (15)	234 (14)	111 (17)	
15–24	957 (13)	673 (14)	212 (13)	72 (11)	
25–44	1901 (27)	1296 (26)	437 (27)	168 (26)	
45–64	1156 (16)	756 (15)	275 (17)	125 (19)	
≥65	571 (8)	326 (7)	198 (12)	47 (7)	

**Patient sex**					
Male	2931 (42)	2036 (42)	648 (41)	247 (39)	0.094
Female	4086 (58)	2761 (58)	937 (59)	388 (61)	

**Aboriginal and/or Torres Strait Islander**					
No	6500 (98)	4446 (98)	1455 (98)	599 (99.3)	0.11
Yes	115 (2)	78 (2)	33 (2)	4 (0.7)	

**NESB**					
No	5974 (90)	4049 (89)	1374 (91)	551 (91)	0.079
Yes	690 (10)	505 (11)	129 (9)	56 (9)	

**Registrar sex**					
Male	3062 (43)	2126 (43)	681 (42)	255 (39)	0.28
Female	4094 (57)	2766 (57)	933 (58)	395 (61)	

**Work hours**					
Part time	1409 (20)	992 (21)	302 (19)	115 (18)	0.59
Full time	5520 (80)	3757 (79)	1249 (81)	514 (82)	

**Registrar training term**					
1	2749 (38)	1861 (38)	642 (40)	246 (38)	0.56
2	3227 (45)	2223 (45)	717 (44)	287 (44)	
3	1180 (16)	808 (17)	255 (16)	117 (18)	

**Worked at practice previously**					
No	5818 (82)	4012 (82)	1295 (81)	511 (80)	0.71
Yes	1277 (18)	853 (18)	300 (19)	124 (20)	

**Qualified as doctor in Australia**					
No	750 (11)	483 (10)	213 (13)	54 (8)	0.011
Yes	6390 (89)	4397 (90)	1397 (87)	596 (92)	

**Practice size**					
Small (1–4 FTE GPs)	2911 (42)	1976 (42)	668 (43)	267 (42)	0.90
Large (≥5 FTE GPs)	4009 (58)	2763 (58)	883 (57)	363 (58)	

**Practice routinely bulk bills**					
No	4235 (62)	2796 (60)	1017 (66)	422 (69)	<0.001
Yes	2609 (38)	1887 (40)	532 (34)	190 (31)	

**Rurality**					
Major city	5157 (73)	3597 (74)	1103 (69)	457 (71)	0.01
Inner regional	1444 (20)	914 (19)	383 (24)	147 (23)	
Outer regional remote	475 (7)	322 (7)	115 (7)	38 (6)	

**Patient/practice status**					
Existing patient	1530 (22)	1045 (22)	361 (23)	124 (19)	0.62
New to registrar	4783 (68)	3266 (68)	1070 (67)	447 (70)	
New to practice	712 (10)	484 (10)	157 (10)	71 (11)	

**Sought help any source**					
No	6284 (88)	4629 (95)	1125 (70)	530 (82)	<0.001
Yes	872 (12)	263 (5)	489 (30)	120 (18)	

**Pathology ordered**					
No	6713 (94)	4653 (95)	1455 (90)	605 (93)	<0.001
Yes	443 (6)	239 (5)	159 (10)	45 (7)	

**Imaging ordered**					
No	7050 (99)	4846 (99.1)	1557 (96)	647 (99.5)	<0.001
Yes	106 (1)	46 (0.9)	57 (4)	3 (0.5)	

**Referral ordered**					
No	7084 (99)	4848 (99.1)	1593 (99)	643 (99)	0.86
Yes	72 (1)	44 (0.9)	21 (1)	7 (1)	

**Follow-up ordered**					
No	4849 (68)	3529 (72)	884 (55)	436 (67)	<0.001
Yes	2307 (32)	1363 (28)	730 (45)	214 (33)	

**Learning goals generated**					
No	6414 (94)	4487 (96)	1342 (87)	585 (94)	<0.001
Yes	393 (6)	165 (4)	192 (13)	36 (6)	

**Respiratory disease**					
Acute bronchitis	734 (10)	237 (5)	417 (26)	80 (12)	<0.001
Acute otitis media	578 (9)	148 (3)	303 (19)	127 (20)	
Acute URTI	4289 (60)	3887 (79)	179 (11)	223 (34)	
Strep/tonsillitis/pharyngitis	1002 (14)	412 (8)	458 (28)	132 (20)	
Acute sinusitis	553 (8)	208 (4)	257 (16)	88 (14)	

**Registrar age, mean (SD)**	32 (5)	31 (5)	32 (6)	31 (4)	0.005

**SEIFA index, mean (SD)**	6 (3)	6 (3)	6 (3)	6 (3)	0.25

**Consultation duration, mean (SD)**	15 (7)	15 (7)	16 (7)	16 (6)	<0.001

**Number of problems/diagnoses, mean (SD)**	2 (1)	2 (1)	1 (1)	1 (1)	<0.001

a *Numbers may not add up to 7156 due to missing data.*

b *Values presented as* n *(%) unless otherwise stated. FTE = full-time equivalent. NESB = non-English-speaking background. SD = standard deviation. SEIFA = Socioeconomic Index for Areas. URTI = upper respiratory tract infection.*

Univariate and multivariable associations of antibiotic prescribing strategies are shown in Table 3. For each association, the OR represents a comparison of immediate prescribing or no prescribing with delayed prescribing. When any in-consultation information or assistance was sought (12% of new ARTI diagnoses; Table 2), no prescribing was less likely (OR 0.41, 95% CI = 0.29 to 0.59) and immediate prescribing was more likely (OR 2.05, 95% CI = 1.48 to 2.84) than delayed prescribing (Table 3). When follow-up was arranged, immediate prescribing was more likely than delayed prescribing (OR 1.31, 95% CI = 1.01 to 1.70). When imaging was ordered, no prescribing was significantly more likely than delayed prescribing (OR 6.26, 95% CI = 1.42 to 27.7); there was some evidence (*P* = 0.068) for immediate prescribing being more likely than delayed prescribing (OR 3.90, 95% CI = 0.90 to 16.9).

**Table 3.** Associations with acute respiratory tract infections^a^

**Variable**	**Outcome class**	**Univariate**		**Adjusted**	

		**OR (95% CI)**	***P*-value**	**OR (95% CI)**	***P*-value**
**Patient age group, years**					
0–<5	No antibiotic	1.19 (0.93 to 1.53)	0.1731	1.98 (1.39 to 2.83)	<0.001
	Immediate prescribing	0.78 (0.59 to 1.04)	0.0869	0.75 (0.51 to 1.10)	0.14
5–14	No antibiotic	0.87 (0.67 to 1.14)	0.3124	1.16 (0.82 to 1.66)	0.40
	Immediate prescribing	0.81 (0.60 to 1.08)	0.1549	0.84 (0.57 to 1.22)	0.36
15–24	No antibiotic	1.25 (0.93 to 1.68)	0.1422	1.35 (0.93 to 1.97)	0.12
	Immediate prescribing	1.17 (0.85 to 1.62)	0.3356	1.25 (0.84 to 1.86)	0.27
45–64	No antibiotic	0.78 (0.61 to 1.01)	0.0575	0.69 (0.50 to 0.95)	0.023
	Immediate prescribing	0.85 (0.64 to 1.12)	0.2515	0.81 (0.57 to 1.15)	0.23
≥65	No antibiotic	0.90 (0.63 to 1.28)	0.5656	0.75 (0.49 to 1.17)	0.21
	Immediate prescribing	1.62 (1.12 to 2.34)	0.0110	1.37 (0.86 to 2.17)	0.19

Aboriginal and/or Torres Strait Islander	No antibiotic	2.81 (1.01 to 7.80)	0.0472	1.73 (0.58 to 5.14)	0.32
	Immediate prescribing	3.11 (1.09 to 8.88)	0.0345	2.58 (0.86 to 7.69)	0.090

Practice routinely bulk bills	No antibiotic	1.54 (1.25 to 1.89)	<0.0001	1.34 (1.03 to 1.74)	0.030
	Immediate prescribing	1.17 (0.94 to 1.46)	0.1534	1.25 (0.95 to 1.64)	0.12

Sought help any source	No antibiotic	0.20 (0.16 to 0.26)	<0.0001	0.41 (0.29 to 0.59)	<0.001
	Immediate prescribing	2.13 (1.68 to 2.69)	<0.0001	2.05 (1.48 to 2.84)	<0.001

Pathology ordered	No antibiotic	0.71 (0.51 to 1.00)	0.0488	0.71 (0.46 to 1.09)	0.12
	Immediate prescribing	1.46 (1.03 to 2.08)	0.0341	1.02 (0.65 to 1.59)	0.93

Imaging ordered	No antibiotic	2.04 (0.63 to 6.63)	0.2380	6.26 (1.42 to 27.7)	0.016
	Immediate prescribing	7.63 (2.37 to 24.6)	0.0007	3.90 (0.90 to 16.9)	0.068

Follow-up ordered	No antibiotic	0.71 (0.59 to 0.86)	0.0003	0.88 (0.68 to 1.13)	0.32
	Immediate prescribing	1.76 (1.44 to 2.15)	<0.0001	1.31 (1.01 to 1.70)	0.044

Learning goals generated	No antibiotic	0.52 (0.35 to 0.77)	0.0010	1.35 (0.79 to 2.29)	0.27
	Immediate prescribing	2.39 (1.64 to 3.50)	<0.0001	1.36 (0.83 to 2.23)	0.23

**Respiratory disease**					
Acute otitis media	No antibiotic	0.06 (0.04 to 0.08)	<0.0001	0.05 (0.04 to 0.07)	<0.001
	Immediate prescribing	3.29 (2.45 to 4.42)	<0.0001	3.04 (2.09 to 4.43)	<0.001
Strep/tonsillitis/pharyngitis	No antibiotic	0.16 (0.13 to 0.21)	<0.0001	0.18 (0.13 to 0.24)	<0.001
	Immediate prescribing	4.85 (3.65 to 6.44)	<0.0001	4.31 (3.04 to 6.11)	<0.001
Acute sinusitis	No antibiotic	0.12 (0.09 to 0.16)	<0.0001	0.17 (0.12 to 0.25)	<0.001
	Immediate prescribing	4.05 (2.94 to 5.59)	<0.0001	3.68 (2.48 to 5.48)	<0.001
Acute bronchitis	No antibiotic	0.15 (0.11 to 0.20)	<0.0001	0.16 (0.11 to 0.24)	<0.001
	Immediate prescribing	6.97 (5.06 to 9.59)	<0.0001	5.93 (4.00 to 8.80)	<0.001

Registrar age, years	No antibiotic	1.02 (1.00 to 1.05)	0.0442	1.05 (1.02 to 1.08)	0.002
	Immediate prescribing	1.04 (1.01 to 1.06)	0.0012	1.03 (1.00 to 1.06)	0.026

Consultation duration, minutes	No antibiotic	0.99 (0.98 to 1.00)	0.0905	0.98 (0.96 to 1.00)	0.019
	Immediate prescribing	1.02 (1.00 to 1.03)	0.0294	0.99 (0.97 to 1.01)	0.19

Number of problems/diagnoses	No antibiotic	1.45 (1.28 to 1.65)	<0.0001	1.87 (1.55 to 2.25)	<0.001
	Immediate prescribing	1.21 (1.05 to 1.39)	0.0088	1.32 (1.08 to 1.60)	0.006

a *The variables patient sex, non-English-speaking background, qualified as doctor in Australia, and rurality were not statistically significantly associated with the outcome and their removal did not alter other coefficients in the model; as such, these covariates were excluded from the multivariable model.*

Longer consultation duration was associated with use of delayed prescribing rather than no prescribing; for each additional minute of consultation duration, the odds of no prescribing decreased by ∼2% (OR 0.98, 95% CI = 0.96 to 1.00). For each additional problem/diagnosis dealt with in the encounter, both no prescribing (OR 1.87, 95% CI = 1.55 to 2.25) and immediate prescribing (OR 1.32, 95% CI = 1.08 to 1.60) were more likely than delayed prescribing. Finally, both no prescribing (OR 1.05, 95% CI = 1.02 to 1.08) and immediate prescribing (OR 1.03, 95% CI = 1.00 to 1.06) were more likely than delayed prescribing with each additional year of age of the GP registrar.

In the post-hoc analyses, when information or assistance was sought from a supervisor (2% of new ARTI diagnoses), immediate prescribing was used significantly more than both delayed prescribing (*P* = 0.004) and no prescribing (*P*<0.001).

## DISCUSSION

### Summary

Early-career GPs use no prescribing of antibiotics for 68% of ARTIs, substantially more than established Australian GPs (43%),^1^ and delayed prescribing for 29% of antibiotic prescriptions for ARTIs. Except for URTIs, they prescribe antibiotics in excess of validated benchmarks.^18^ Prescribing antibiotics was associated with markers of diagnostic uncertainty such as seeking in-consultation assistance and arranging follow-up.

### Strengths and limitations

A high response rate, which is unusual for studies of GPs,^19^ along with little missing data on antibiotic prescribing (0.3%) are strengths of this study. GP registrar characteristics were reflective of the Australian GP registrar population overall,^20^ and their practices were located in major cities, inner and outer regional areas, and remote areas. The participating Regional Training Organisations train 43% of Australian GP registrars.^20^ That a large proportion of Australian registrars participated in the study, and their characteristics were similar to those of the wider registrar population, suggests generalisability to Australian specialist general practice vocational training.

A limitation is that contextual clinical details for the presenting conditions are not known, so it was not possible to judge how appropriate the prescription of antibiotics was for any individual problem/diagnosis; however, the finding that total prescribing is greatly in excess of benchmarks for all ARTIs other than URTI is robust because clinical judgement is implicit to the range of those benchmarks.^18^ A risk of misclassification bias and social desirability bias does exist, however, when a registrar does not accurately record an ARTI diagnosis because they prescribed antibiotics; the authors regard this risk as likely to be small — in ReCEnT, registrars record the broad range of their clinical activities over consecutive consultations (there is no focus on any single activity, including antibiotic prescribing).

The authors did not have data on whether patients filled the delayed prescriptions or actually took the dispensed antibiotics, nor did they have data on clinical outcomes; however, this is not considered a substantive limitation as the focus of the study was registrars’ prescribing behaviour. To put the findings into some context, however, it has been found in randomised controlled trials of delayed prescribing that 31% of delayed scripts will be filled (with delayed scripts provided at consultation being more likely to be filled than those to be collected later).^5^

### Comparison with existing literature

In the study presented here, GP registrars used no prescribing substantially more often than established Australian GPs in McCullough *et al*’s modelling^1^ for acute bronchitis/bronchiolitis (32% versus 15%), otitis media (26% versus 11%), and sore throat (41% versus 6%) (Figure 1, Supplementary Table S1). For URTI, GP registrars prescribed within the benchmark specified by the European Surveillance of Antimicrobial Consumption disease-specific benchmarks,^18^ which have been validated for use in Australian general practice,^21^ but this was not the case for the other ARTIs reported here.

When prescribing antibiotics, it appears Australian GP registrars are using delayed prescribing more often than European GPs: in a multi-country study, European GPs used delayed prescribing for 12% of prescriptions written for lower respiratory tract infections/ARTIs with cough as the dominant symptom.^9^ This percentage is most closely comparable with acute bronchitis/bronchiolitis in the study presented here — in which registrars used delayed prescribing for 16% of prescriptions written. For all cases (including where no antibiotic was prescribed), European GPs used delayed prescribing in 6.3%,^9^ compared with 11% of bronchitis/bronchiolitis in the study presented here.

Prescribing antibiotics was more likely when there were markers of clinical concern, such as seeking information or assistance during the consultation or arranging specific follow-up. This suggests GP registrars may be addressing their diagnostic uncertainty regarding a more serious illness and the perceived consequences of not prescribing antibiotics. The authors have previously found that, when GP registrars seek help from their supervisor, they are significantly more likely to prescribe antibiotics for URTI and acute bronchitis^22^ — diagnoses for which authoritative Australian guidelines recommend not prescribing antibiotics.^23^ Data for this study do not include clinical information to ascertain how appropriate antibiotic prescription was in other ARTIs, but it is noted that established GPs prescribe substantially more antibiotics for ARTIs in Australia than GP registrars^1^ and also prescribe more than in similar medical systems in Europe and Canada.^2^ Consequently, one possibility is that GP registrars may use immediate prescribing to be consistent with a supervisor’s and/or a practice-wide approach to ARTIs; this is consistent with the authors’ findings of qualitative research in this area^24^ and may be supported by the finding of GP registrars being more likely to prescribe an antibiotic if they ask their supervisor for information or assistance.

Ordering imaging (for example, chest X-ray) can also be interpreted as a marker of clinical concern, but it is strongly associated with no prescribing. In Australia, GPs (including GP registrars) can order imaging, including a radiologist report, and expect it to be performed the same day. One interpretation of this association could relate to advice to confirm a possible pneumonia diagnosis with a chest X-ray,^23^ resulting in a delay in initiating antibiotics at first presentation; consequently, the GP registrar may diagnose an ARTI and investigate further before deciding whether it is pneumonia. The authors also found some evidence for immediate prescribing also being associated with imaging; this is consistent with the GP registrar, in some cases, addressing their diagnostic uncertainty by taking a pre-emptive approach and deciding to prescribe antibiotics for a more-severe ARTI, whether or not a subsequent chest X-ray demonstrates pneumonia.^24^

Delayed prescribing is associated with longer consultation duration; this may reflect more time being needed for complex or concerning presentations, with diagnostic uncertainty leading to the ‘safety net’ of delayed antibiotic prescribing. This is consistent with the authors’ qualitative findings in this area,^24^ but it may be that more time is needed to undertake an explanation of delayed prescribing rather than no prescribing.

The finding that with each additional problem/diagnosis dealt with in the consultation, delayed prescribing was increasingly less likely than both no prescribing and immediate prescribing could suggest that the GP registrar is choosing a strategy that requires less explanation and, consequently, less time in a busy consultation.

These associations with prescribing antibiotics — that is, markers of clinical concern, increased consultation duration, fewer problems/diagnoses managed per consultation — provide limited evidence that is congruent with qualitative findings. Some GP registrars use delayed prescribing, not only to address their own diagnostic uncertainty, but also to accommodate conflicting influences on prescribing for ARTIs such as: national guideline advice against use of antibiotics, desire to adhere to antimicrobial stewardship, knowledge that delayed prescribing reduces antibiotic consumption, both perceived and actual expectation from patients that antibiotics are necessary, and a supervisor approach or practice culture to prescribing for ARTIs.^22^^,^^24^

The associations of the individual respiratory infective illness classifications used in this study, when compared with URTI, showed immediate prescribing was more likely than delayed prescribing, and delayed prescribing was more likely than no prescribing. The findings regarding otitis media, sore throat, and sinusitis may partly reflect Australian guideline recommendations^23^ that antibiotics are indicated for these diagnoses in selected situations (and that delayed prescribing is an option in some circumstances). However, the associations of acute bronchitis (and the large effect sizes of these associations) cannot be reconciled with current Australian guideline recommendations that antibiotics are not indicated for acute bronchitis.^23^

### Implications for practice

Delayed prescribing represents a viable method for changing primary care practice to reduce the consumption of antibiotics in the community and is already being used quite frequently by early-career GPs in Australia. As GP registrars represent 13% of the Australian GP workforce by headcount,^20^^,^^25^ the findings have immediate, as well as medium- and long-term, implications for the quality of general practice care. Efforts to increase the use of both delayed prescribing and no prescribing for ARTIs are indicated, including in GP training programmes. In particular, encouraging the use of delayed prescribing may enable an acceptable transition to a future environment of more-rational antibiotic prescribing for ARTIs in primary care.
